# Knockdown of circ_0055412 promotes cisplatin sensitivity of glioma cells through modulation of CAPG and Wnt/β‐catenin signaling pathway

**DOI:** 10.1111/cns.13820

**Published:** 2022-03-25

**Authors:** Qingjiu Zhou, Qiang Fu, Mahati Shaya, Yalikun Kugeluke, Shaoshan Li, Yisireyili Dilimulati

**Affiliations:** ^1^ Department of Neurosurgery the First Affiliated Hospital of Xinjiang Medical University Urumqi Xinjiang China; ^2^ Department of Oncology the First Affiliated Hospital of Xinjiang Medical University Urumqi Xinjiang China

**Keywords:** CAPG, circ_0055412, glioma, NFATC3, Wnt/β‐catenin signaling pathway

## Abstract

**Introduction:**

Glioma is the most frequent primary cerebral tumor in adults. Recent evidence has suggested that circular RNAs (circRNAs) are associated with the pathological processes in glioma. In our study, we aimed to investigate the function and mechanism of circ_CAPG (circ_0055412) in glioma.

**Methods:**

Firstly, circ_0055412 expression was examined through RT‐qPCR analysis. Loss‐of‐function assays and animal experiments were implemented to evaluate the role of circ_0055412 on cisplatin resistance of glioma cells. Moreover, mechanism assays were done to probe into the regulatory mechanism of circ_0055412 in glioma cells.

**Results:**

Circ_0055412 was found to be notably upregulated in glioma cells. Moreover, depletion of circ_0055412 enhanced cisplatin sensitivity of glioma cells in vitro and in vivo. Moreover, circ_0055412 recruited eukaryotic translation initiation factor 4A3 (EIF4A3) protein to stabilize capping actin protein, gelsolin like (CAPG) mRNA. Furthermore, circ_0055412 served as a sponge for microRNA‐330‐3p (miR‐330‐3p) and regulated nuclear factor of activated T cells 3 (NFATC3) expression to activate the transcription of catenin beta 1 (CTNNB1), thus participating in the activation of Wnt/β‐catenin signaling pathway.

**Conclusion:**

Circ_0055412 contributed to cisplatin resistance of glioma cells via stabilizing CAPG mRNA and modulating Wnt/β‐catenin signaling pathway. This finding might provide novel information for the treatment of glioma.

## Graphical abstract

The scientific concept map describes the functional mechanism of circ_0055412 in the cisplatin sensitivity of glioma cells.

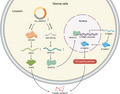

## 1 INTRODUCTION

Glioma is derived from astrocytes, oligodendrocytes, or their precursors, which accounts for 81% of malignant brain tumors.^1^ Although most glioma subtypes are relatively rare, some glioma subtypes cause high mortality.^2^ Among gliomas, glioblastoma represents 45% of all gliomas and is the most malignant form.^3^ Although advances in glioma treatment have been achieved, including radiotherapy, chemotherapy, and surgery, the overall survival rate for glioblastoma patients remains low.^4^ Moreover, tumors also change and adapt in response to drugs.^5^ To improve the situation, seeking for methods to enhance the chemotherapy sensitivity of glioma cells is especially important.

Up to date, cisplatin (DDP), a widely used chemotherapeutic agent, has been applied for the treatment of various kinds of tumors.^6^ Although DDP has great significance in improving the therapeutic effect of cancers, the existence of resistance to DDP may lead to therapeutic failure.^7^ According to previous studies, the application of DDP has been proved to play the antitumor role in glioma.^8^ However, for glioblastoma, its heterogeneity is also correlated to the resistance to chemotherapy, which makes the treatment of glioblastoma more difficult.^9^ Hence, to improve the therapeutic effect of glioblastoma, exploring potential factors, which may influence the resistance to DDP is urgent.

Circular RNAs (circRNAs) are covalently closed, endogenous transcripts, which are characterized by covalently linked 3′–5′ ends. As a main type of non‐coding RNAs, circRNAs are commonly considered to be crucial participators in tumors.^10^ For example, Zhu et al. have manifested that circRNA_100533 hinders the process of oral squamous cell carcinoma via the modulation of miR‐933/GNAS.^11^ Tang et al. have proposed that circ_0000515 accelerates the development of cervical cancer via working as a competing endogenous RNA (ceRNA).^12^ Lu et al. have demonstrated that circ‐RanGAP1 influences the invasion and metastasis of gastric cancer through elevating VEGFA expression.^13^ In addition, the impact of circRNAs on the cisplatin resistance or sensitivity of cancer cells has also been deeply investigated. For instance, Guan et al. have elucidated that circRNA_102272 enhances cisplatin resistance of hepatocellular carcinoma cells via sponging miR‐326 and regulating RUNX2 expression.^14^ Mao et al. have confirmed that circRNA CDR1‐AS influences cisplatin resistance in lung adenocarcinoma through the activation of EGFR/PI3K pathway.^15^ Liu et al. have disclosed that circ_0060060 affects cisplatin resistance in thyroid carcinoma through serving as a sponge for miR‐144‐3p.^16^

Wnt/β‐catenin signaling pathway has been widely identified to be implicated in cancers.^17^ The link between circRNAs and Wnt/β‐catenin signaling pathway has been also highlighted in cancers.^18^ For example, Zhang et al. have attested circRNA_069718 mediates the progression of triple‐negative breast cancer through the activation of Wnt/β‐catenin pathway.^19^ Huang et al. have uncovered that circRNA_104348 activates Wnt pathway to deteriorate hepatocellular carcinoma development.^20^

Circ_0055412 is a circRNA cyclized from capping actin protein, gelsolin like (CAPG). In this study, we targeted at examining the expression of circ_0055412 in glioma cells and assessing the role of circ_0055412 in the cisplatin sensitivity of glioma cells. Moreover, how circ_0055412 exerts its roles in glioma also needs to be explored.

## 2 MATERIALS AND METHODS

### 2.1 Cell culture

Glioma cells (H4, A172, U251, and LN229), normal human glial cell line (HEB), and HEK‐293T cells were used in this research. H4, A172, LN229 along with HEK‐293T cells were procured from ATCC. U251 and HEB cells were bought from Huatuo Biotechnology Co., Ltd. The cells grew in Dulbecco's modified eagle's medium (DMEM) with 10% FBS. Cell culture plates were kept in a humid environment at 37°C with 5% CO_2_.

### 2.2 Cell transfection

The short hairpin RNAs (shRNAs) used to downregulate circ_0055412, EIF4A3 or NFATC3 were devised and synthesized by RiboBio. MiR‐330‐3p mimics/inhibitor as well as corresponding negative control (NC) was procured from RiboBio. Furthermore, pcDNA3.1 vectors employed to overexpress CAPG, NFATC3 or CTNNB1 along with the empty pcDNA3.1 was prepared. The 48‐h plasmid transfection was completed with the help of lipofectamine 3000 (Invitrogen).

### 2.3 Quantitative reverse transcription polymerase chain reaction (RT‐qPCR)

Trizol (Invitrogen) was employed to achieve total RNA extraction. RNA concentration was analyzed with the use of NanoDrop 2000 (Thermo Scientific). PrimeScript™ RT master mix (Takara, Japan) was applied to synthesize cDNA. PCR was completed utilizing SYBR Green PCR Master Mix (Applied Biosystems). GAPDH and U6 were used for internal references of cytoplasm and nucleus, severally. Relative expression of genes was measured via 2^−ΔΔCT^ method. Assay was performed in triplicate.

### 2.4 Cell Counting Kit 8 (CCK‐8) assay

Cells were placed into 96‐well plates (5 × 10^3^ cells/well). To test the cisplatin sensitivity, the IC50 value was measured according to the reported literature.^21^ This experiment was independently implemented in triplicate.

### 2.5 5‐Ethynyl‐2′‐deoxyuridine (EdU) assay

EdU detection kit (RiboBio) was applied for assessing cell proliferative ability. Cells were cultivated in 96‐well plates, followed by the treatment of 100 µl of 50 µM EdU for 2 h. Then cells were treated with 4% paraformaldehyde and 0.5% Triton X‐100, followed by the treatment of 100 µl of 1× Apollo^®^ 488 fluorescent staining reaction. DAPI was utilized to conduct cell staining and the cells were visualized with the help of a fluorescence microscope. This experiment was independently implemented in triplicate.

### 2.6 Flow cytometry analysis

After glioma cells were transfected for 48 h, 70% precooling ethanol was applied to fix cells at 4°C for 12 h. Next, cells were centrifugated. After being rinsed with PBS, cells were incubated with 100 μl PI dye and 100 μl RNA enzymes, followed by slow and full precipitation. Cell apoptosis was observed by a flow cytometer. The experiment was independently conducted in triplicate.

### 2.7 Transferase‐mediated dUTP nick end labeling (TUNEL) assay

Under the guidance of TUNEL assay kit (Beyotime), cells were mixed with TUNEL reaction mixture in the dark for 1 h after indicated fixation and permeabilization. After DAPI staining, fluorescent microscope was utilized. The experiment was carried out for at least three times.

### 2.8 Subcellular fractionation assay

By means of cytoplasmic and Nuclear RNA Purification Kit (Norgen), cytoplasm, and nucleus of glioma cells were separated. The expression of circ_0055412 was examined by RT‐qPCR. GAPDH or U6 was, respectively, deemed as the internal reference of cytoplasm or nucleus. This experiment was independently conducted in triplicate.

### 2.9 Fluorescent in situ hybridization (FISH) and immunofluorescence (IF)

After being fixed in 4% paraformaldehyde and permeabilized by 0.5% Triton X‐100, glioma cells were incubated with circ_0055412 FISH probe in hybridization buffer. Cells were next treated with DAPI and a confocal laser microscope (Olympus) was eventually applied for taking pictures.

For IF analysis, anti‐EIF4A3 primary antibody was co‐cultured with glioma cells overnight at 4°C. Subsequently, FITC‐conjugated secondary antibody was added for incubation. Similarly, images were captured. The experiment was performed in triplicate.

### 2.10 RNA pull down assay

Glioma cell lysate was incubated with biotinylated circ_0055412 probes and magnetic beads. The target RNAs or proteins enriched in complexes and collected by beads were eluted for western blot or RT‐qPCR detection. This experiment was performed in triplicate.

### 2.11 RNA immunoprecipitation (RIP)

Under the instruction of the manual of Z‐Magna RIP™ RNA‐binding protein immunoprecipitation kit (Millipore Corporation), RIP assay was done. Anti‐Ago2 (Abcam), anti‐EIF4A3 (Abcam) as well as anti‐IgG (Abcam) were added into cell lysates for incubation. Finally, the RNA‐protein complexes were purified and subjected to RT‐qPCR analysis. This experiment was performed in triplicate.

### 2.12 Chromatin immunoprecipitation (ChIP)

The EZ ChIP™ Chromatin Immunoprecipitation Kit (Millipore) was applied for completing ChIP assay. Firstly, DNA fragments were obtained after sonication. Next, anti‐NFATC3 (Abcam) or anti‐IgG was added for co‐cultivation. Eventually, the immunoprecipitated DNA was subject to qPCR detection. The assay was independently carried out in triplicate.

### 2.13 Luciferase reporter assay

To verify the interaction between CTNNB1 promoter and NFATC3, the CTNNB1 promoter covering the wild type (Wt) or mutant type (Mut) binding sites was inserted into pGL3 vector (Promega), and the successfully constructed plasmids were co‐transfected with pcDNA3.1/NFATC3 or empty pcDNA3.1 into glioma cells. In a similar manner, pmirGLO‐circ_0055412‐Wt, pmirGLO‐circ_0055412‐Mut, pmirGLO‐NFATC3 3′UTR‐Wt or pmirGLO‐NFATC3 3′UTR‐Mut was severally co‐transfected with miR‐330‐3p mimics or NC mimics. For circ_0055412‐related pathway, cells were transfected with various luciferase reporter constructs (NOTCH pathway, Wnt pathway, PI3K/AKT pathway, Hedgehog pathway, MAPK pathway, and NF‐κB pathway) and sh‐circ_0055412#1/2. After transfection for 48 h, the luciferase activity was examined through a Luciferase Reporter Assay System (Promega). This experiment was performed in triplicate.

### 2.14 TOP/FOP flash assay

Glioma cells were transfected with sh‐NC, sh‐NFATC3#1 or sh‐NFATC3#2 as well as TOP/FOP‐Flash. With dual‐luciferase reporter assay Kit (Promega), luciferase activities were detected. This assay was implemented in triplicate.

### 2.15 Tumor xenograft assay

Qualified mice (4 weeks old) were bought from Institute of model animals, Nanjing University, and divided into four groups (eight mice for each group), and then severally injected with cells transfected with sh‐NC and sh‐circ_0055412#1 plasmids with the presence or absence of DDP. About 5 × 10^6^ cells were inoculated subcutaneously into mice after mice accommodated in the new environment for 1 week. Tumor volume was recorded every 3 days. Finally, mice were sacrificed after 27 days, and the tumor weight was measured after tumor excision. The Ethics Committee of the First Affiliated Hospital of Xinjiang Medical University approved this study.

### 2.16 Western blot analysis

Glioma cells were lysed, and the proteins were collected. Next, the protein samples were separated and moved to PVDF membranes (Millipore). After that, the membranes sealed by non‐fat milk were incubated with primary antibodies of CAPG, EIF4A3, Nuclear β‐catenin, Histone H3, c‐myc, cyclin D1, NFATC3, CTNNB1, β‐actin, and GAPDH. Later, secondary antibody was added. Eventually, Chemiluminescence system (GE Healthcare) was operated and protein expression was quantified. This experiment was performed in triplicate.

### 2.17 Statistical analysis

Data acquired from experiments were analyzed by SPSS 22.0 statistical software package and displayed as mean ± standard deviation (SD). The statistic difference between two or more groups was analyzed by means of Student's *t*‐test or ANOVA. The abovementioned experiments were independently carried out in triplicate. Differences were deemed to have statistical significance when *p* < 0.05. Shapiro‐Wilk Test was utilized to assess data distribution.

## 3 RESULTS

### 3.1 The characterization of circ_0055412

**FIGURE 1:**
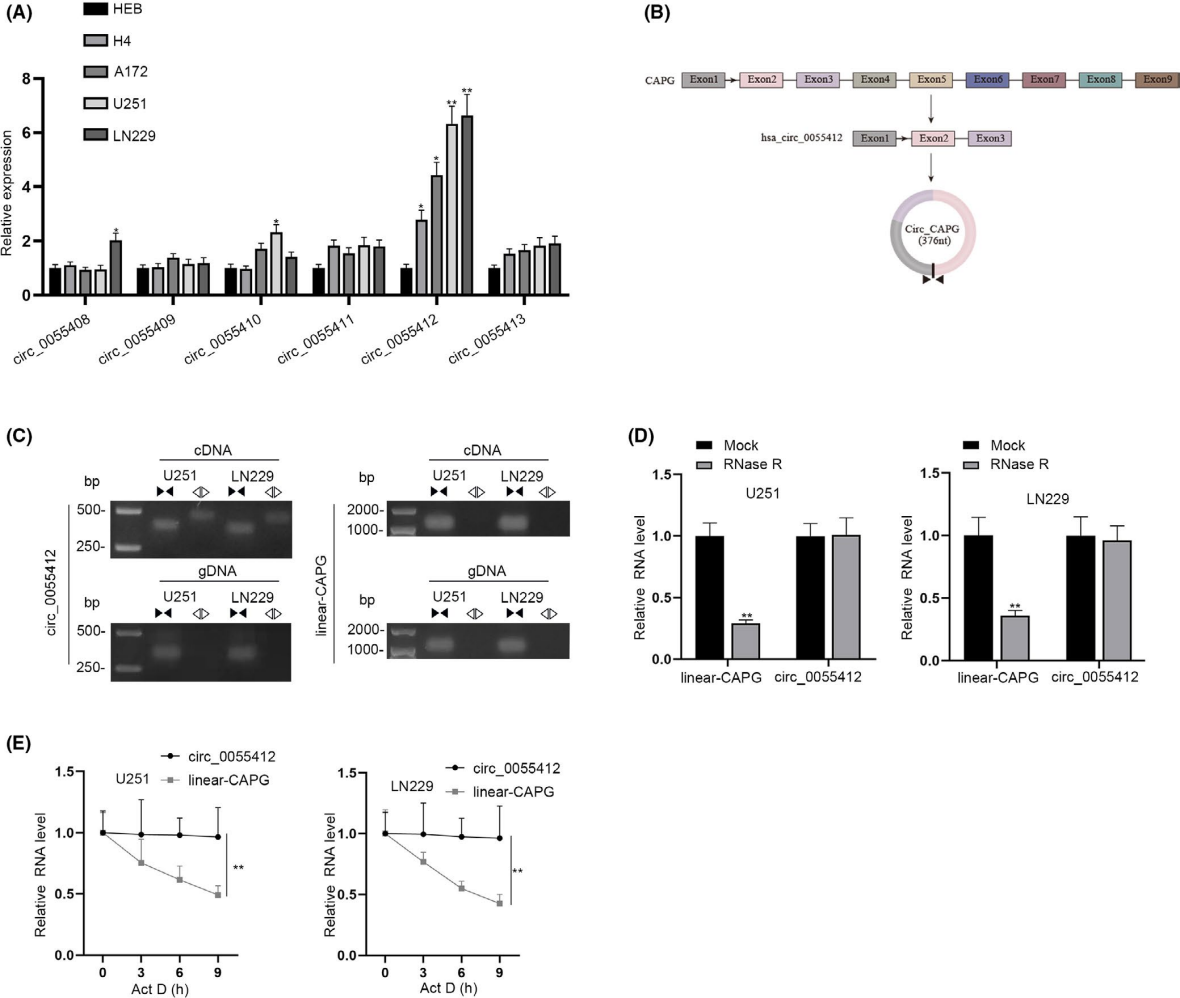
Circ_0055412 is significantly high expressed in glioma cells and features as the stable loop structure. (A) RT‐qPCR was performed to quantify the expression of six circRNAs cyclized from CAPG in glioma cell lines and normal cell line. (B) Schematic illustrated the generation of circ_0055412. (C) Gel electrophoresis was employed to test the structure of circ_0055412 with the help of divergent primers of cDNA. (D) RT‐qPCR was carried out to evaluate the stability of circ_0055412 and linear‐CAPG expression in glioma cells treated with RNase R. (E) The stability of circ_0055412 and linear CAPG in glioma cells was tested after the treatment of Act D. *p < 0.05, **p < 0.01

According to a previous study, CAPG has been demonstrated to be high in glioma, and it is connected with the severity of the cancer as well as patients’ prognosis.^22^ Therefore, in this study, we speculated that circRNAs, which were cyclized from CAPG might also influence glioma progression a. Firstly, we detected the expression of all six circRNAs (circ_0055408, circ_0055409, circ_0055410, circ_0055411, circ_0055412, and circ_0055413) cyclized from CAPG, noticing that circ_0055412 expression was notably high in glioma cell lines (H4, A172, U251, and LN229) compared with normal cell line (HEB) (Figure 1A). The schematic diagram of the genomic location of circ_0055412 was demonstrated in Figure 1B. To investigate the structure of circ_0055412, the convergent and divergent primers were specifically designed to amplify the linear and back‐splicing forms of CAPG. The data gained from PCR displayed that circ_0055412 were only amplified by divergent primers using cDNA templates instead of genomic DNA (gDNA) templates (Figure 1C). RT‐qPCR analysis also revealed that linear CAPG was digested while circ_0055412 was resistant to RNase R degradation after glioma cells were treated with RNase R (Figure 1D). After adding actinomycin D (Act D) to inhibit RNA synthesis, we also discovered that circ_0055412 was more stable than linear CAPG (Figure 1E). In conclusion, circ_0055412 displays markedly high expression in glioma cells.

### 3.2 Silencing of circ_0055412 enhances the cisplatin sensitivity of glioma cells in vitro and in vivo

**FIGURE 2:**
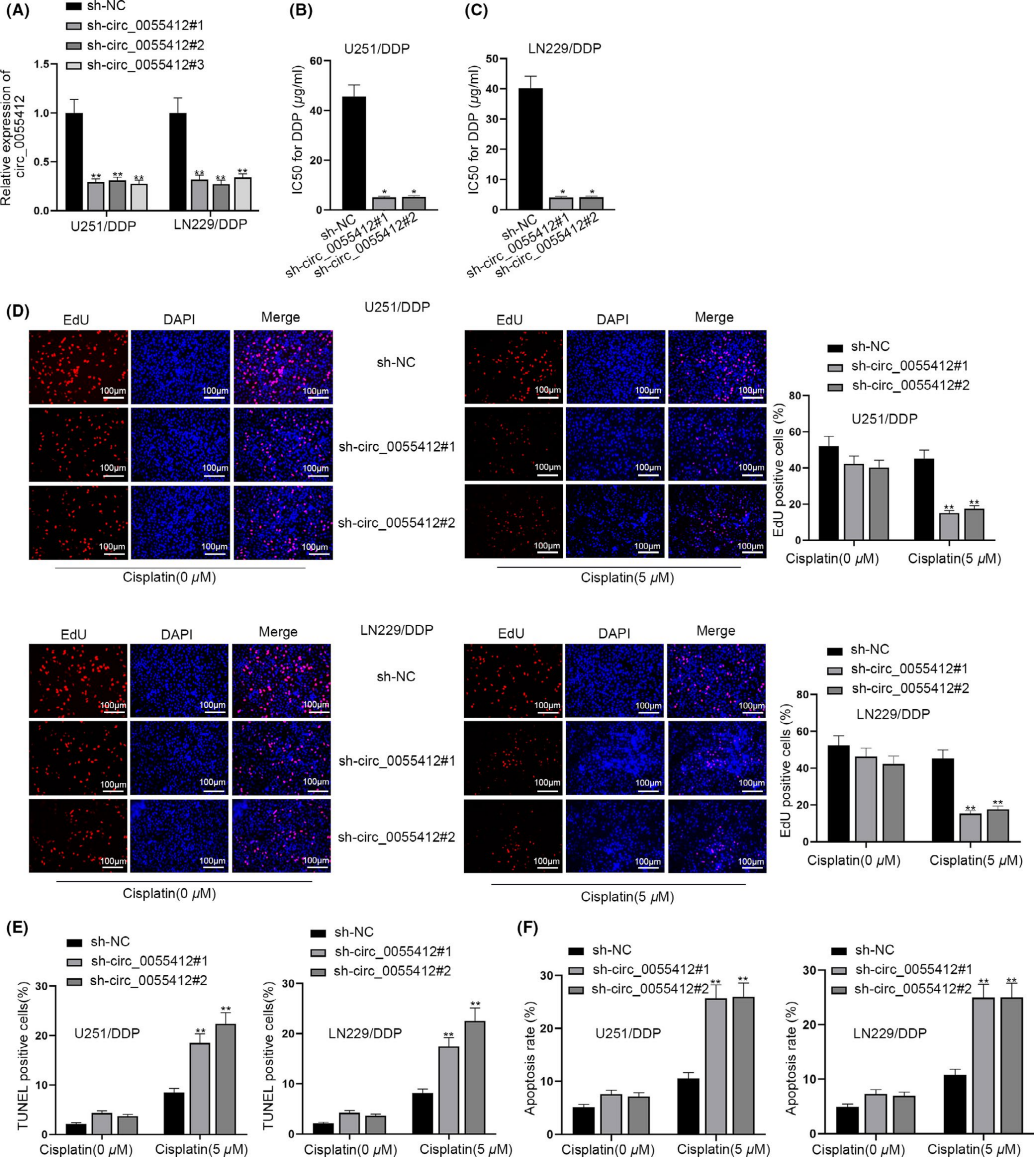
Depletion of circ_0055412 enhances the cisplatin sensitivity of glioma cells in vitro and in vivo. (A) The knockdown efficacy of sh‐circ_0055412#1/2/3 was evaluated by RT‐qPCR in U251/DDP and LN229/DDP cells. (B, C) CCK‐8 assay was conducted to examine the change of DDP resistance in DDP‐resistant U251 and LN229 cells after circ_0055412 was silenced. (D) EdU assay was performed to examine cell proliferation with or without the treatment of DDP. (E, F) The apoptotic rate of DDP‐resistant U251 and LN229 cells was evaluated by TUNEL assay and flow cytometry analysis. *p < 0.05, **p < 0.01

Accumulating evidence has demonstrated that circRNAs may affect cisplatin resistance or sensitivity in cancer cells.^23^, ^24^ Hence, we made a conjecture that circ_0055412 might also be concerned with cisplatin sensitivity in glioma. To testify the conjecture, we firstly treated U251 and LN229 cells with DDP and conducted CCK‐8 assay to test IC50 for DDP. The results suggested that the IC50 value was significantly higher in cisplatin‐resistant U251/DDP and LN229/DDP cells than that in cisplatin‐sensitive U251 and LN229 parental cells (Figure S1A,B). RT‐qPCR also disclosed a notably enhanced expression of circ_0055412 in U251/DDP and LN229/DDP cells compared with parental U251 and LN229 cells, which indicated that circ_0055412 might modulate the cisplatin sensitivity of glioma cells (Figure S1C). Subsequently, functional assays were performed. Firstly, circ_0055412 expression was cut down in DDP‐resistant U251 and LN229 cells (Figure 2A). The experimental outcomes of CCK‐8 assay manifested that when circ_0055412 was downregulated, the IC50 value of U251/DDP and LN229/DDP cells was also decreased (Figure 2B,C). EdU proliferation assay uncovered that with the presence of cisplatin (5 μM), circ_0055412 depletion significantly hampered proliferation of DDP‐resistant U251 and LN229 cells (Figure 2D). Conversely, from TUNEL assay and flow cytometry analysis, we observed that after the treatment of cisplatin (5 μM), inhibition of circ_0055412 strengthened the apoptotic capacity of U251/DDP and LN229/DDP cells (Figure 2E,F). Further, animal experiments were conducted to assess the influence of circ_0055412 on cisplatin sensitivity in glioma *in vivo*. As exhibited in Figure S2A, the tumor growth rate became much slower in sh‐NC +cisplatin (5 μM) group compared to sh‐NC+cisplatin (0 μM). Moreover, in sh‐circ_0055412#1+cisplatin (5 μM) group, tumor weight became lighter in comparison with the control group sh‐NC+cisplatin (5 μM) (Figure S2B). To be summarized, circ_0055412 contributes to cisplatin resistance of glioma cells.

### 3.3 Circ_0055412 modulates CAPG expression in glioma cells

**FIGURE 3:**
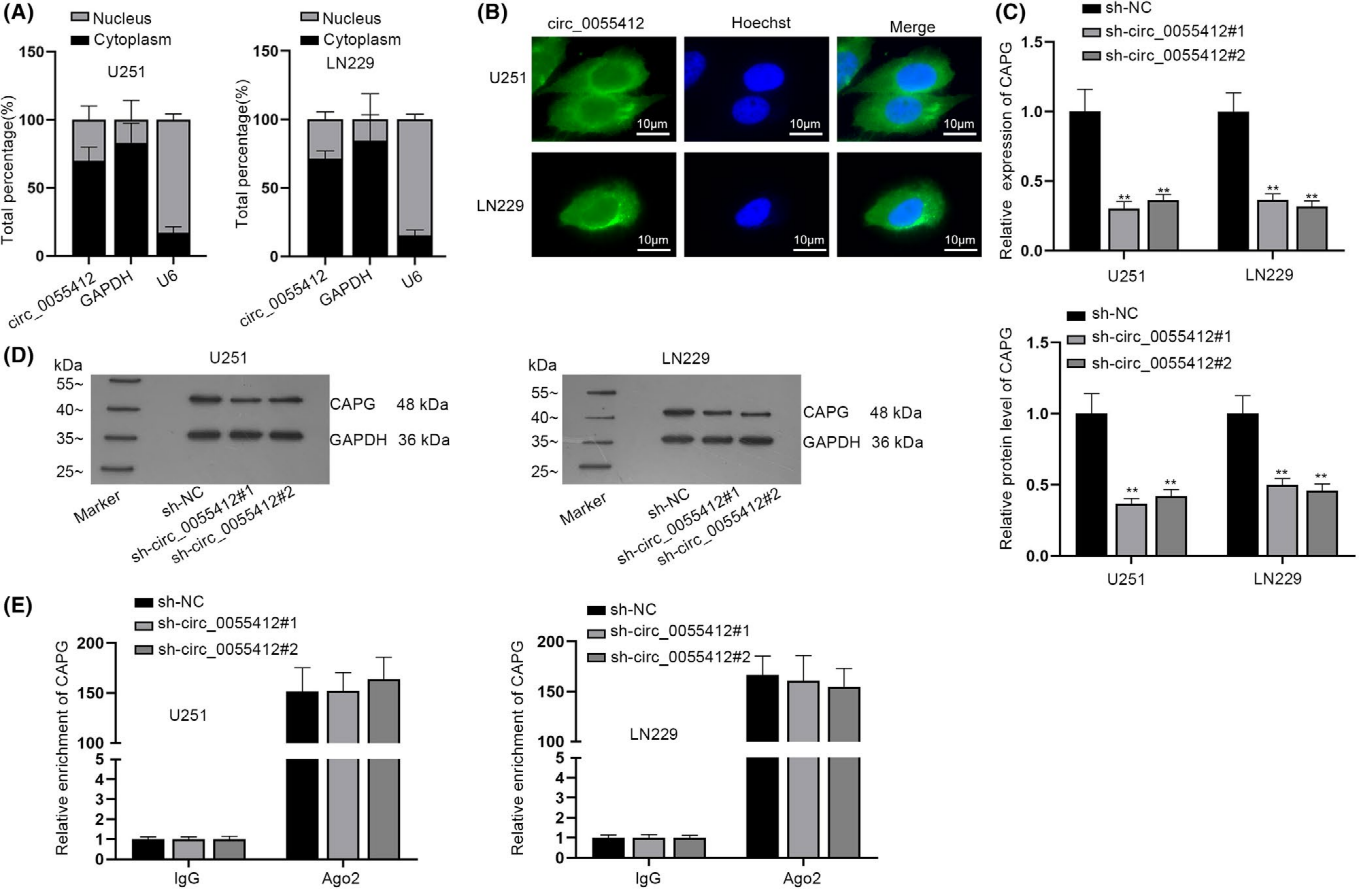
Circ_0055412 positively modulates CAPG expression in glioma cells. (A, B) The localization of circ_0055412 in glioma cells was investigated by subcellular fractionation and FISH assays. (C, D) CAPG expression at mRNA and protein levels was examined when circ_0055412 expression was lessened in glioma cells. (E) RIP assay was operated to examine the enrichment of CAPG in Ago2 antibody when circ_0055412 was downregulated. **p < 0.01

To get a better understating of the latent regulatory mechanism of circ_0055412 in glioma cells, subcellular fractionation, and FISH assays were firstly implemented. It turned out that circ_0055412 was mainly located in the cytoplasm of glioma cells (Figure 3A,B). At the same time, RT‐qPCR and Western blot analyses represented that circ_0055412 interference led to the decline of CAPG expression at mRNA and protein levels (Figure 3C,D). Furthermore, RIP assay validated that CAPG was enriched in the Ago2 antibody and when circ_0055412 was silenced, no evident change was observed in the enrichment of CAPG in the Ago2 antibody (Figure 3E), which excluded the existence of ceRNA mechanism between circ_0055412 and CAPG. In a word, CAPG is positively regulated by circ_0055412.

### 3.4 Circ_0055412 recruits EIF4A3 protein to stabilize CAPG mRNA

**FIGURE 4:**
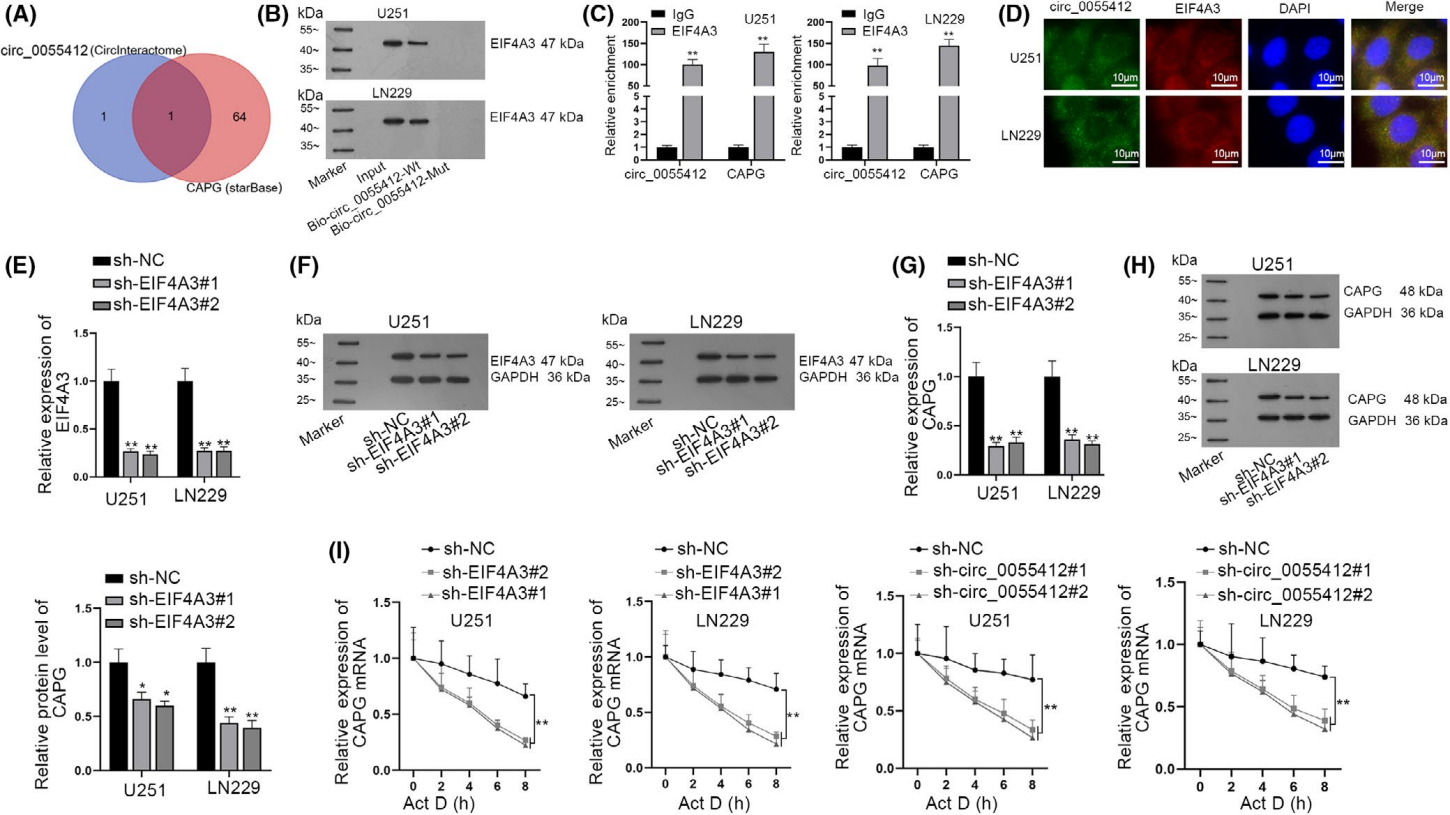
Circ_0055412 recruits EIF4A3 protein to enhance the stability of CAPG mRNA. (A) The overlapping results predicted from circinteractome and starBase databases were exhibited by Venn diagram. (B) RNA pull down assay was carried out to explore the combination of circ_0055412 with EIF4A3. (C) RIP assay was performed to assess the interaction between EIF4A3 and circ_0055412 or CAPG. (D) FISH and IF analyses were implemented to detect the localization of circ_0055412 and EIF4A3 in glioma cells. (E, F) The mRNA and protein levels of EIF4A3 were detected with the use of RT‐qPCR and western blot assays in U251 and LN229 cells upon EIF4A3 deficiency. (G, H) CAPG expression was tested via RT‐qPCR and western blot assays after EIF4A3 was depleted in glioma cells. (I) CAPG mRNA stability was examined through RT‐qPCR when EIF4A3 or circ_0055412 was downregulated in glioma cells. *p < 0.05, **p < 0.01

Based on the previous findings acquired from Figure 3E, we assumed that circ_0055412 might regulate CAPG expression through interacting with certain protein. With the application of circinteractome (https://circinteractome.nia.nih.gov/) and starBase (https://circinteractome.nia.nih.gov/), EIF4A3 was selected (Figure 4A). The strong affinity of circ_0055412 and EIF4A3 was exhibited through RNA pull down assay (Figure 4B). Meanwhile, RIP assay displayed that circ_0055412 and CAPG were highly enriched in the EIF4A3 antibody (Figure 4C). Also, FISH assay and IF analysis showed that circ_0055412 and EIF4A3 were both accumulated in the cytoplasmic part of U251 and LN229 cells (Figure 4D). Subsequently, we lessened EIF4A3 expression at mRNA and protein levels by transfecting sh‐EIF4A3#1/2 plasmids (Figure 4E,F) and discovered that CAPG expression was accordingly reduced after depletion of EIF4A3 (Figure 4G,H). Moreover, the stability of CAPG mRNA was reduced due to downregulation of EIF4A3 or circ_0055412 (Figure 4I). To get a better understanding of the relationship between circ_0055412 and CAPG in the cisplatin sensitivity of glioma cells, rescue assays were carried out. Through the transfection of pcDNA3.1/CAPG plasmid, CAPG was upregulated in U251/DDP cells (Figure S3A). The results of EdU assay mirrored that under the condition of cisplatin (5 μM), the inhibited cell proliferation imposed by circ_0055412 interference was partially restored by CAPG upregulation (Figure S3B). On the contrary, cell apoptosis assays implied that with the presence of cisplatin (5 μM), circ_0055412 silencing suppressed cell apoptosis was partially regained by overexpression of CAPG (Figure S3C,D). Collectively, circ_0055412 stabilizes CAPG mRNA by interacting with EIF4A3 protein.

### 3.5 Circ_0055412 serves as a sponge for miR‐330‐3p

**FIGURE 5:**
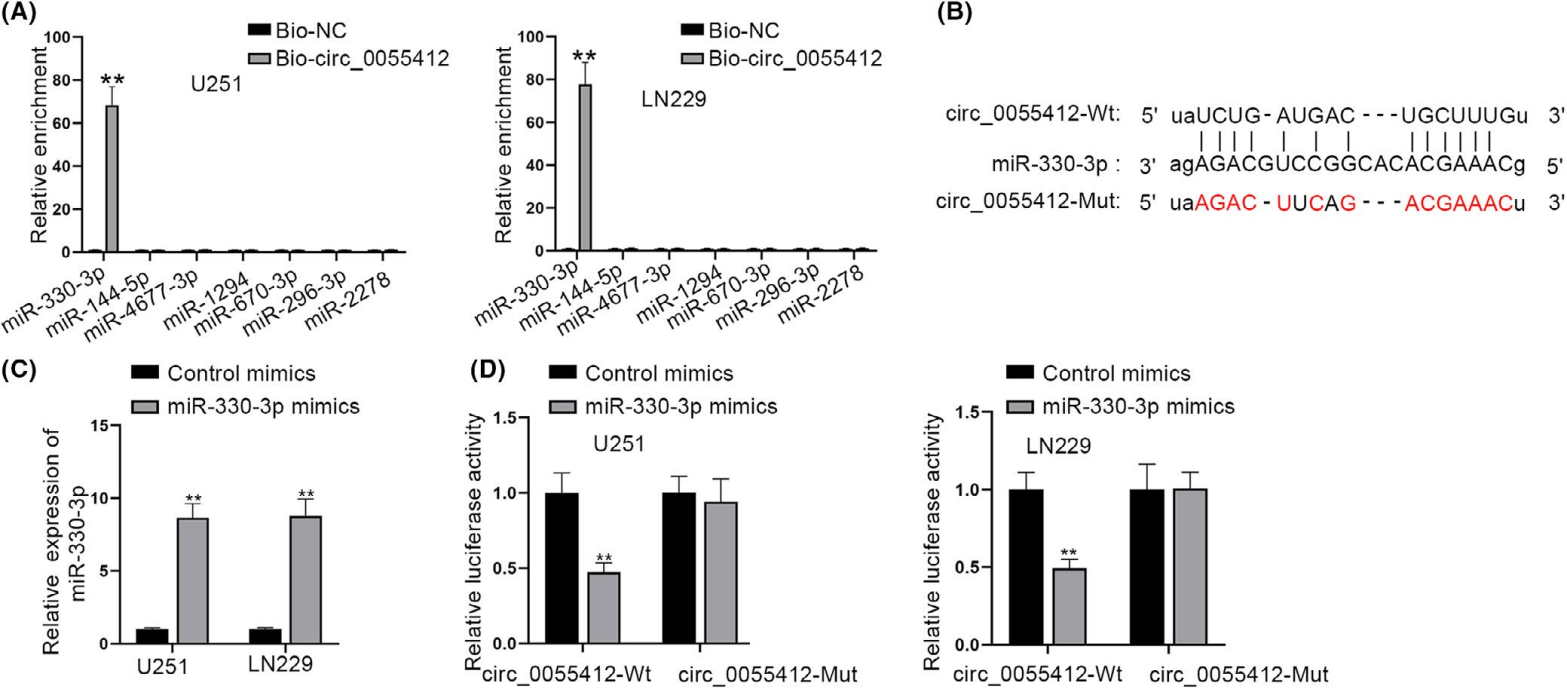
Circ_0055412 serves as a sponge for miR‐330‐3p. (A) RNA pull down assay was operated to examine the enrichment of predicted 7 miRNAs in the biotin‐labeled circ_0055412 probe. (B) Binding regions between circ_0055412 and miR‐330‐3p was demonstrated. (C) The efficiency of miR‐330‐3p overexpression was examined by RT‐qPCR in glioma cells. (D) The luciferase activities in the circ_0055412‐Wt group and circ_0055412‐Mut group with miR‐330‐3p upregulation were checked by luciferase reporter assay. **p < 0.01

According to the previous findings of Figure 3A,B, we speculated that circ_0055412 might exert its roles at post‐transcriptional level through ceRNA mechanism. With the application of starBase, we predicted 7 potential miRNAs (miR‐330‐3p, miR‐144‐5p, miR‐4677‐3p, miR‐1294, miR‐670‐3p, miR‐296‐3p and miR‐2278) of circ_0055412. Further, RNA pull down assay verified that among these miRNAs, and only miR‐330‐3p was preferentially accumulated in the biotin‐labeled circ_0055412 probe (Figure 5A). The predicted binding sequences between circ_0055412 and miR‐330‐3p were manifested in Figure 5B. After confirming the high overexpression efficacy of miR‐330‐3p (Figure 5C), we implemented luciferase reporter assay. Upregulation of miR‐330‐3p was found to cause a decrease of the luciferase activity in the circ_0055412‐Wt group (Figure 5D). In summary, miR‐330‐3p is sequestered by circ_0055412.

### 3.6 Circ_0055412 modulates NFATC3 expression through sequestering miR‐330‐3p

**FIGURE 6:**
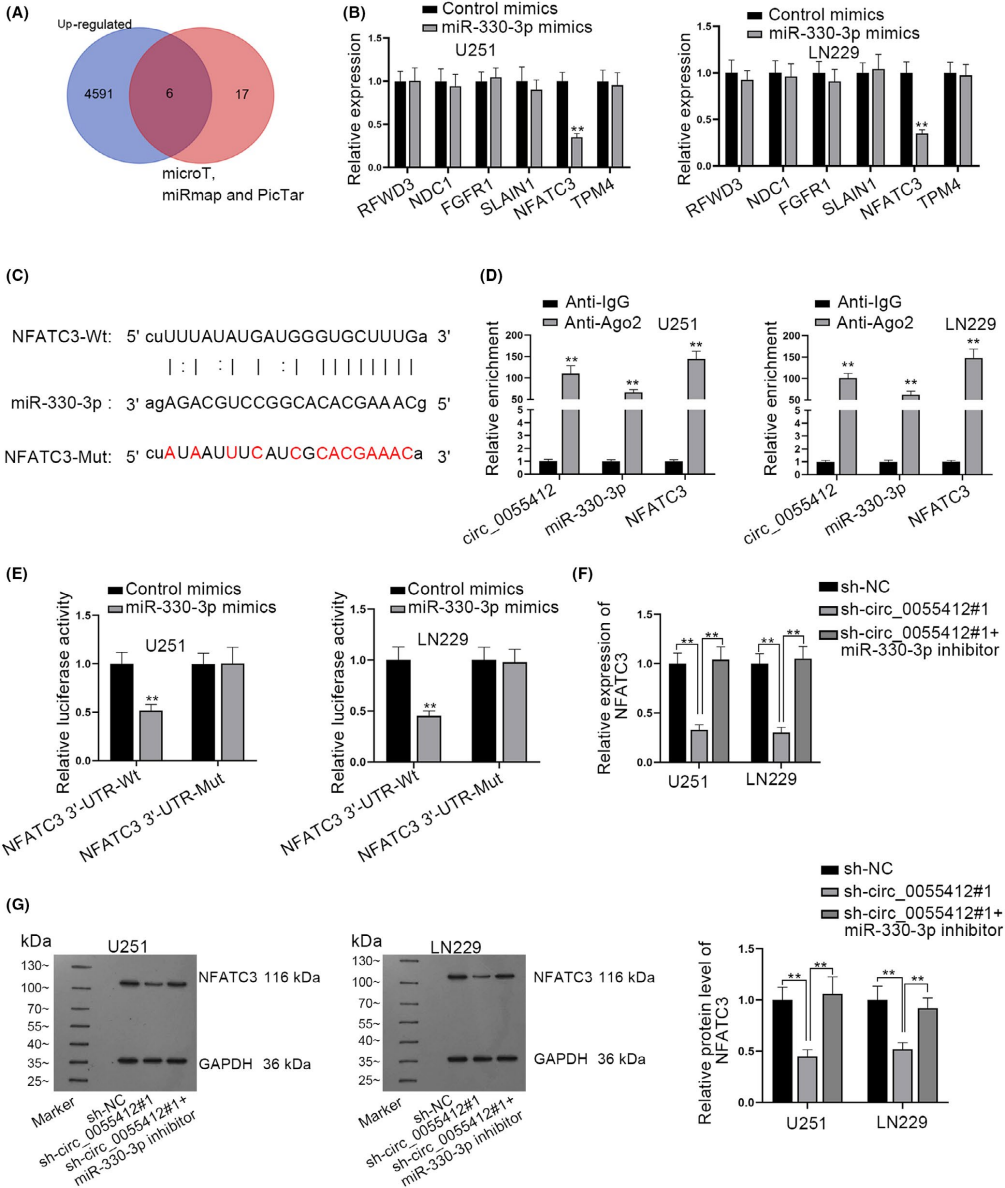
Circ_0055412 modulates NFATC3 expression through sequestering miR‐330‐3p. (A) Potential target genes of miR‐330‐3p were demonstrated via Venn diagram. (B) The expression of predicted 6 mRNAs was analyzed through RT‐qPCR when miR‐330‐3p was overexpressed. (C) Binding sequences between miR‐330‐3p and NFATC3 was displayed. (D) RIP assay was carried out to evaluate the binding relationship among circ_0055412, miR‐330‐3p and NFATC3. (E) The binding between miR‐330‐3p and NFATC3 was confirmed by luciferase reporter assay. (F‐G) The mRNA and protein level of NFATC3 was analyzed in the sh‐NC group, sh‐circ_0055412#1 group and sh‐circ_0055412#1+miR‐330‐3p inhibitor group. **p < 0.01

Following, to determine the target gene of miR‐330‐3p, we utilized GEPIA (http://gepia.cancer‐pku.cn/detail.php) and starBase and predicted 6 potential mRNAs (RFWD3, NDC1, FGFR1, SLAIN1, NFATC3 and TPM4) (Figure 6A). RT‐qPCR analyzed that only NFATC3 expression displayed a significant decline in response to miR‐330‐3p upregulation (Figure 6B). As demonstrated in Figure 6C, the binding sequences between miR‐330‐3p and NFATC3 were predicted. The enrichment of circ_0055412, miR‐330‐3p and NFATC3 in Anti‐Ago2 was exhibited by RIP assay (Figure 6D). Luciferase reporter assay also revealed the binding between miR‐330‐3p and NFATC3 (Figure 6E). Finally, RT‐qPCR and western blot analysis reflected that the decrease in NFATC3 expression resulting from circ_0055412 interference was completely restored on account of miR‐330‐3p inhibition (Figure 6F,G).

### 3.7 NFATC3 is the transcription activator of CTNNB1 promoter

**FIGURE 7:**
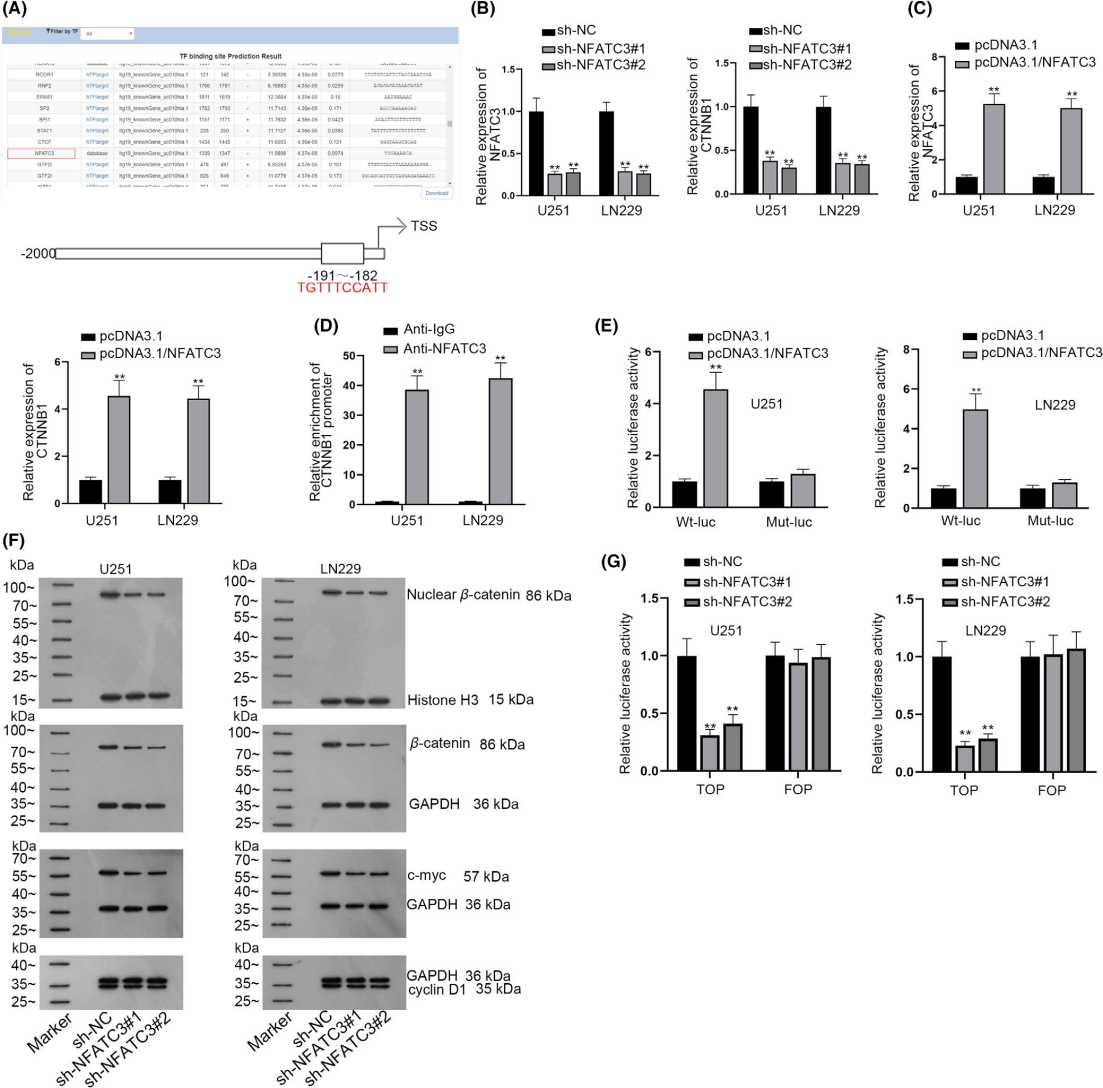
NFATC3 is the transcription activator of CTNNB1 promoter. (A) UCSC, Human TFDB and JASPAR were applied to predict candidate transcription factors of CTNNB1. (B) The knockdown efficacy of sh‐NFATC3#1/2 was tested, and CTNNB1 expression was detected when NFATC3 was downregulated through RT‐qPCR assay. (C) The overexpression efficiency of pcDNA3.1‐NFATC3 was examined and the expression of CTNNB1 was detected in CTNNB1‐upregulated glioma cells through RT‐qPCR assay. (D) ChIP assay was conducted to investigate the binding affinity between NFATC3 and CTNNB1 promoter. (E) Luciferase reporter assay tested the luciferase activity of CTNNB1 promoter (Wt or Mut) when NFATC3 was overexpressed in glioma cells. (F) Western blot was done to explore the protein levels of nuclear β‐catenin, CTNNB1, c‐myc and cyclin D1 after NFATC3 was inhibited. (G) The luciferase activity of Wnt/β‐catenin signaling pathway was examined with the use of TOP/FOP flash assay when NFATC3 expression was reduced in glioma cells. **p < 0.01

It is reported that the correlation between circRNAs and Wnt/β‐catenin signaling pathway has great significance in the onset and progression of cancers.^18^ Via luciferase reporter assay, we discovered that circ_0055412 insufficiency decreased the luciferase activity of Wnt signaling pathway instead of NOTCH pathway, PI3K/AKT pathway, Hedgehog pathway, MAPK pathway and NF‐kB pathway (Figure S4A). Western blot further presented that the protein levels of nuclear β‐catenin, c‐myc and cyclin D1 were significantly decreased in response to circ_0055412 deficiency (Figure S4B). Additionally, CTNNB1 expression was cut down after circ_0055412 was depleted (Figure S4C). Furthermore, we intriguingly observed that the luciferase activity of CTNNB1 promoter was also reduced when circ_0055412 was silenced (Figure S4D). Thence, we assumed that circ_0055412 might mediate the transcription of CTNNB1 to activate the Wnt/β‐catenin signaling pathway. With the application of UCSC (http://genome.ucsc.edu/index.html), Human TFDB (http://bioinfo.life.hust.edu.cn/HumanTFDB#!/) and JASPAR (http://jaspar.genereg.net/) database, we discovered that NFATC3 was one of the transcription factors of CTNNB1 (Figure 7A). Also, CTNNB1 expression was decreased when NFATC3 was downregulated while CTNNB1 expression was increased when NFATC3 was upregulated (Figure 7B,C). The interaction between NFATC3 and CTNNB1 promoter was also validated by ChIP and luciferase reporter assays (Figure 7D,E). Further, western blot analysis manifested that NFATC3 depletion reduced the protein levels of nuclear β‐catenin, CTNNB1, c‐myc and cyclin D1 (Figure 7F). TOP/FOP flash reporter assay also uncovered that inhibition of NFATC3 decreased the activity of Wnt/β‐catenin signaling pathway (Figure 7G). To confirm whether circ_0055412 enhanced cisplatin resistance of glioma cells via enhancing CTNNB1 expression, rescue assays were conducted. CTNNB1 was overexpressed in U251/DDP cells after transfection of pcDNA3.1/CTNNB1 (Figure S5A). Cell proliferation assay elucidated that upregulation of CTNNB1 partially counteracted the inhibited cell proliferation imposed by circ_0055412 deficiency with the presence of cisplatin (5 μM) (Figure S5B). TUNEL assay and flow cytometry analysis also revealed that under the condition of cisplatin (5 μM), circ_0055412 knockdown promoted cell apoptosis while CTNNB1 overexpression partially countervailed this effect (Figure S5C,D). Briefly, on one hand, circ_0055412 recruited EIF4A3 protein to stabilize CAPG mRNA. On the other hand, circ_0055412 sequestered miR‐330‐3p and regulated NFATC3 expression to promote the transcription of CTNNB1, which enhanced β‐catenin and activated Wnt/β‐catenin pathway, thereby improving the cisplatin resistance of glioma cells.

## 4 DISCUSSION

It is commonly acknowledged that circRNAs act as important regulators in malignant tumors,^10^ glioma is also included.^25^ For example, Ding et al. have unmasked that circNFIX plays the promoting role in glioma by sponging miR‐378e and modulating RPN2 expression.^26^ Cui et al. have demonstrated that circ_0005075 induces cell proliferation and migration in glioma by decreasing SIRT1 expression.^27^ Meanwhile, a large amount of evidence has determined that circRNAs mediate the cisplatin resistance or sensitivity of cancer cells.^23^, ^24^ As reported, CAPG is related to proliferation, metastasis and prognosis in glioma.^28^ Furthermore, a previous study has proved that CAPG contributes to cell proliferation in glioma.^28^ In our study, we discovered that circ_0055412, which was derived from CAPG was dramatically overexpressed in glioma cells and concerned with cisplatin resistance of glioma cells. Functional assays and animal experiments demonstrated that interference of circ_0055412 facilitated the cisplatin sensitivity of glioma cells in vitro and in vivo.

As the host gene of circ_0055412, CAPG was discovered to be positively modulated by circ_0055412. Through subcellular fractionation and FISH assays, the cytoplasmic location of circ_0055412 in glioma cells was exhibited. Hence, we inferred that circ_0055412 modulate regulate CAPG expression at post‐transcriptional level. CeRNA and RNA‐binding protein (RBP) are identified to be two main mechanisms in post‐transcriptional events.^29^, ^30^ RIP assay unveiled that the enrichment of CAPG in Ago2 antibody was not influenced by circ_0055412 knockdown, which excluded the ceRNA mechanism. Therefore, we made a conjecture that circ_0055412 regulated CAPG expression via recruiting certain proteins. EIF4A3 has been reported to be capable of maintaining the stability of mRNAs.^31^ In addition, Wang et al. have revealed that EIF4A3 activates the transcription of circMMP9 in glioblastoma.^32^ Through our investigation, we found that circ_0055412 stabilized CAPG mRNA through interacting with EIF4A3 protein. Moreover, upregulation of CAPG partially restored the enhanced cisplatin sensitivity of glioma cells induced by silencing of circ_0055412.

Subsequently, we assumed that circ_0055412 might influence the cisplatin resistance of glioma cells through another mechanism. It has been reported that miR‐330‐3p suppresses the process of many cancers, such as liver cancer,^33^ colorectal cancer^34^ and gastric cancer.^35^ At the same time, miR‐330‐3p has been identified to work as a tumor suppressor in glioma.^36^, ^37^ Consistent with these findings, miR‐330‐3p was testified to be sequestered by circ_0055412. NFATC3 has been demonstrated to play the oncogenic role in oral squamous cell carcinoma.^38^ Furthermore, it is implicated that NFATC3 influences tumor growth in human astroglioma.^39^ Similarly, we validated that NFATC3 was a target gene of miR‐330‐3p. Furthermore, upregulation of NFATC3 partially recovered the enhanced cisplatin sensitivity of glioma cells caused by circ_0055412 depletion.

Emerging evidence has presented that Wnt/β‐catenin signaling pathway engages in diverse cellular activities in glioma whose activation is characterized by the import of β‐catenin into the nucleus.^40^ Our findings attested that NFATC3 activated the transcription of CTNNB1 to increase β‐catenin and stimulate Wnt/β‐catenin signaling pathway.

In conclusion, our study elucidates the regulatory mechanism that circ_0055412 regulated EIF4A3/CAPG axis and miR‐330‐3p/NFATC3/Wnt/β‐catenin pathway to promote cisplatin resistance of glioma cells, which might provide the theoretical and practical basis for glioma therapy. However, our study also has some limitations. We do not investigate the regulatory mechanism about upregulation of circ_0055412 in glioma cells. As a result, more studies are needed to thoroughly elucidate the molecular cross‐talk involved.

## CONFLICT OF INTEREST

No conflicts of interest exist.

## Supporting information

Supplementary Material

## Data Availability

Data sharing not applicable to this article as no datasets were generated or analysed during the current study.
