# Supplementary material for: Knockdown of circ_0055412 promotes cisplatin sensitivity of glioma cells through modulation of CAPG and Wnt/β‐catenin signaling pathway
Source: CNS Neurosci Ther. 2022 Mar 25;28(6):884–96. doi: 10.1111/cns.13820 (PMC9062567; doi:10.1111/cns.13820)

Full unedited gel for Figure 1C

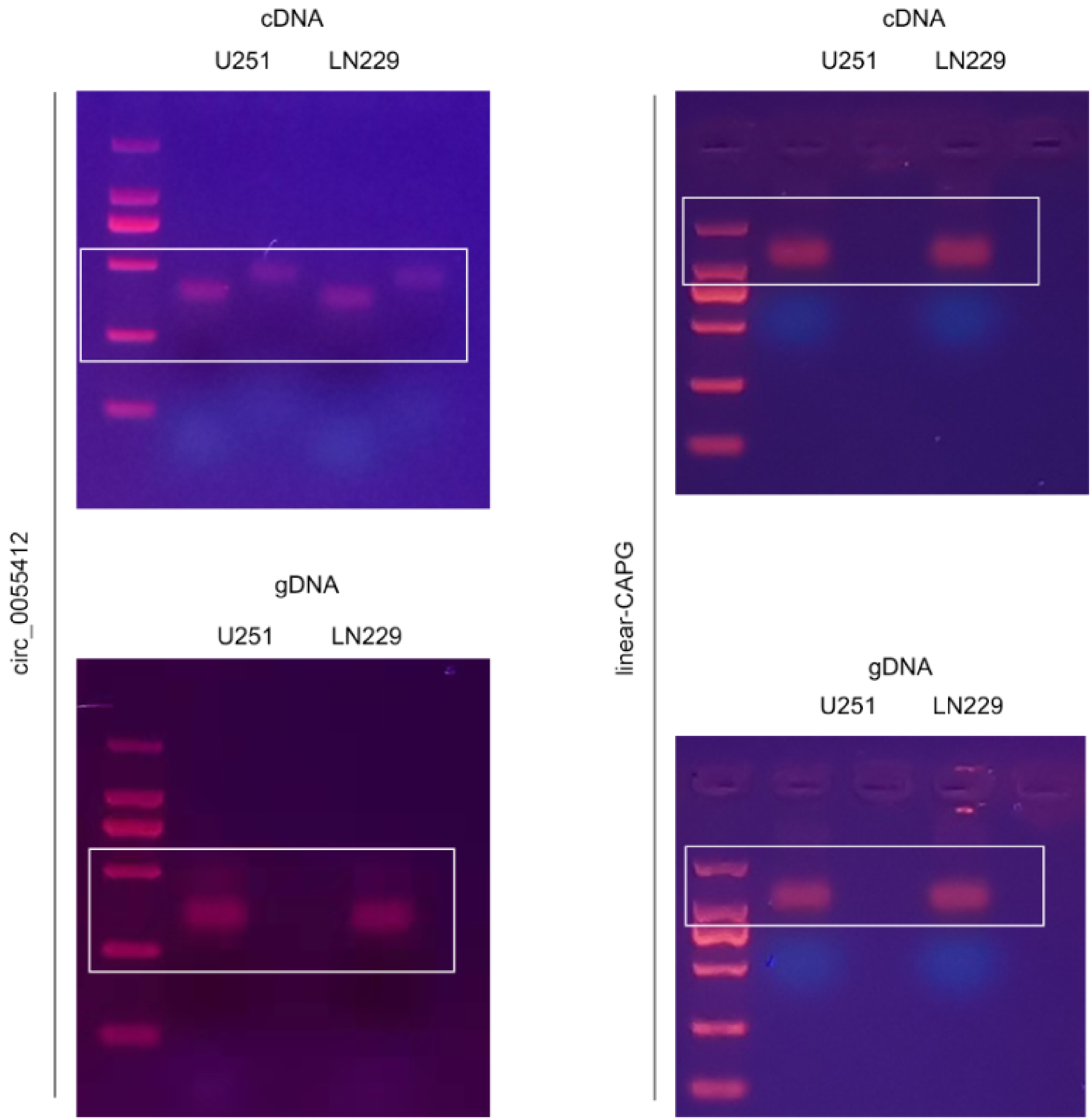

Full unedited blot for Figure 3D

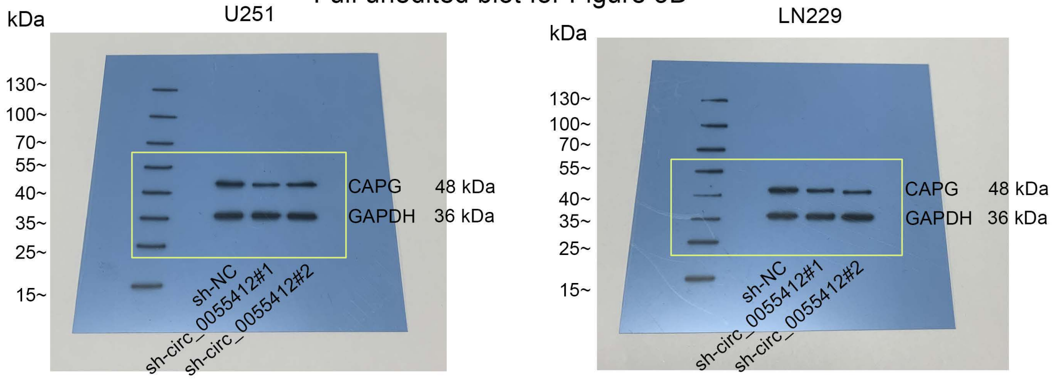

Full unedited blot for Figure 4B

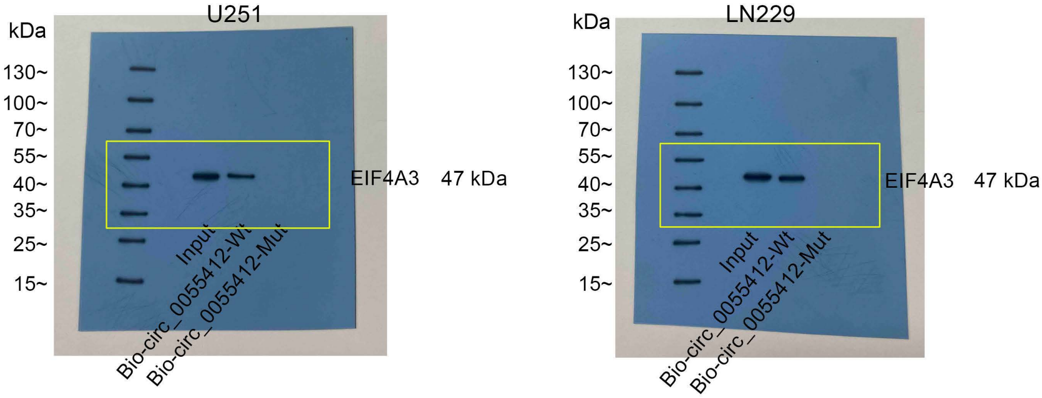

Full unedited blot for Figure 4F

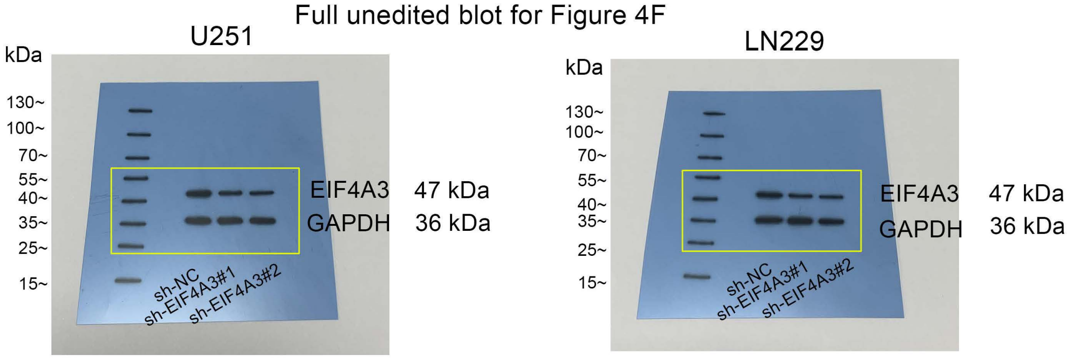

Full unedited blot for Figure 4H

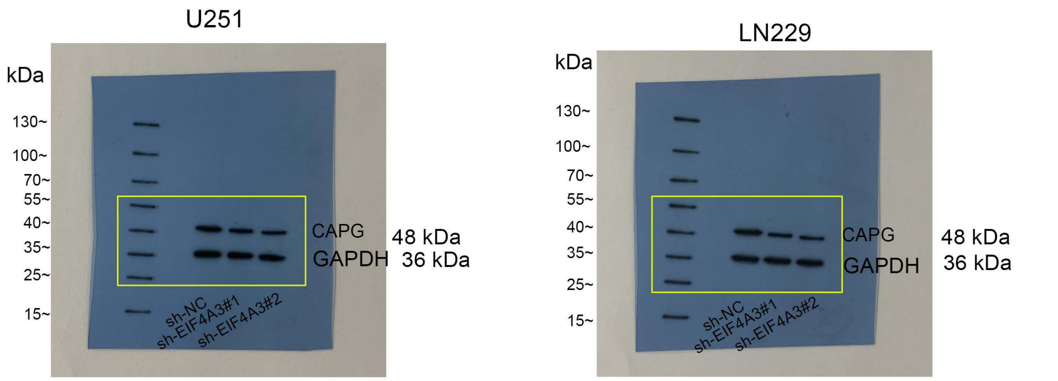

Full unedited blot for Figure 6G

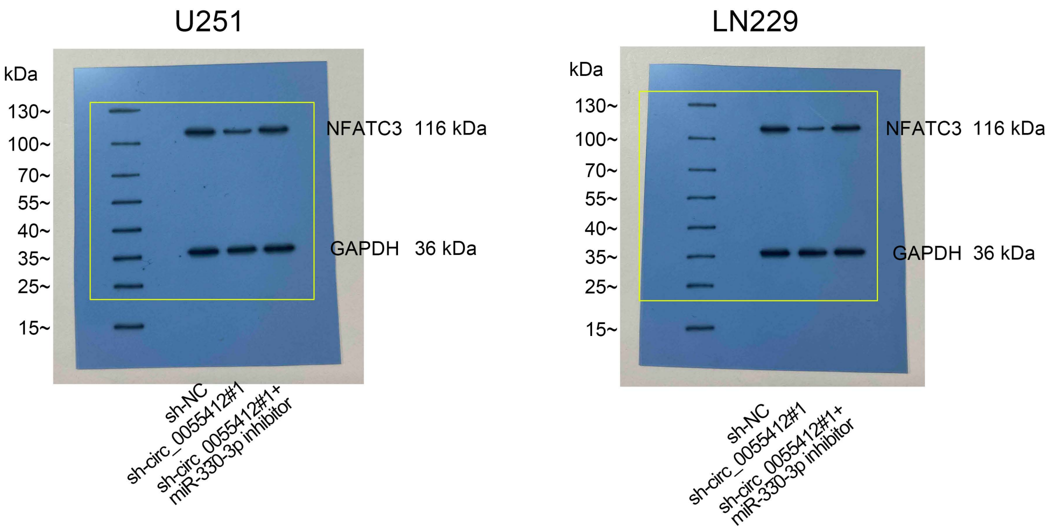

Full unedited blot for Figure 7F

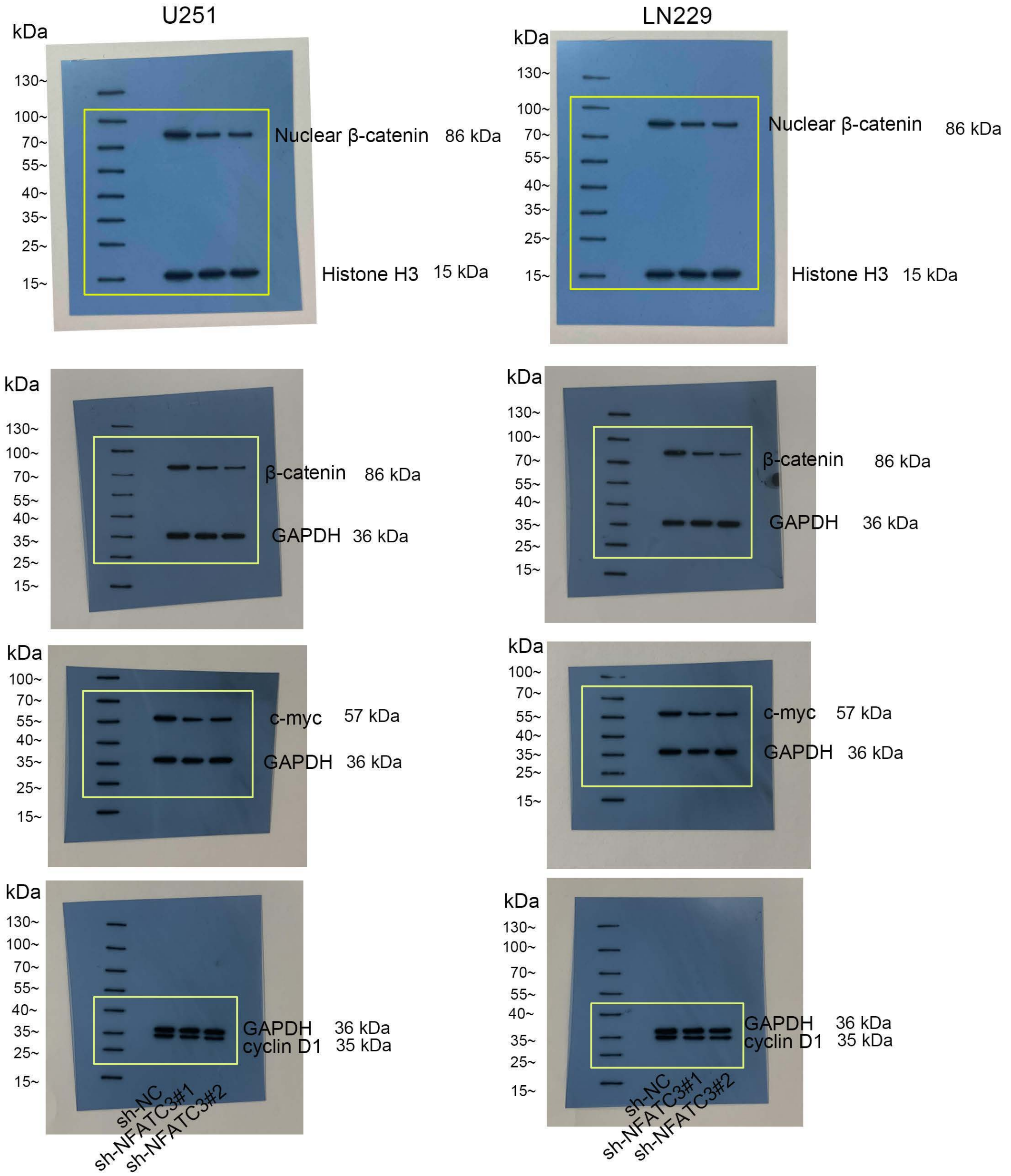

Full unedited blot for Figure S4B

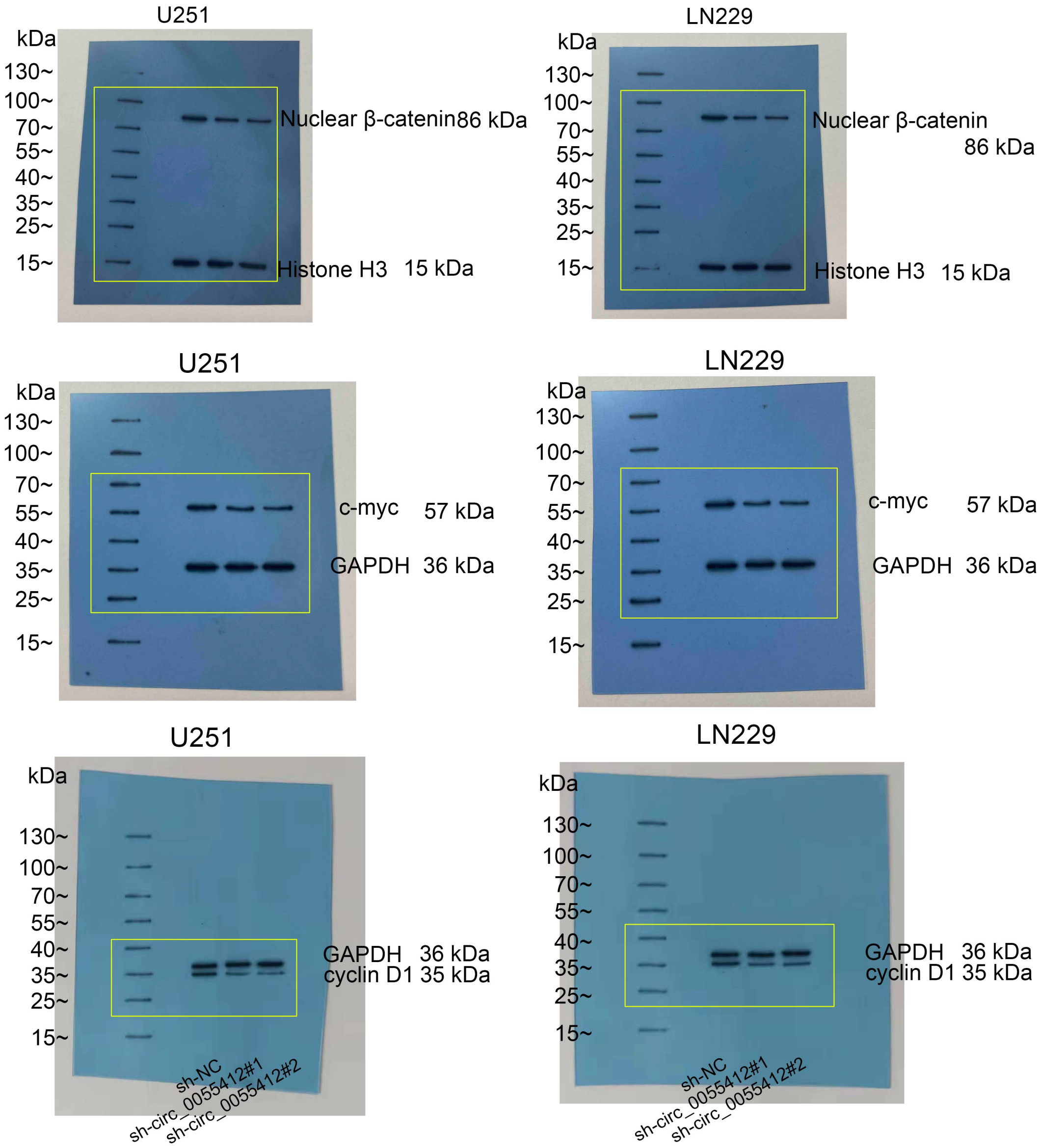

Supplement: Supplementary file 1 — Supplementary Material [file CNS-28-884-s001.zip › cns13820-sup-0002-Fig1C.pdf]
