# Supplementary figures and images for: Knockdown of circ_0055412 promotes cisplatin sensitivity of glioma cells through modulation of CAPG and Wnt/β‐catenin signaling pathway
Source: CNS Neurosci Ther. 2022 Mar 25;28(6):884–96. doi: 10.1111/cns.13820 (PMC9062567; doi:10.1111/cns.13820)

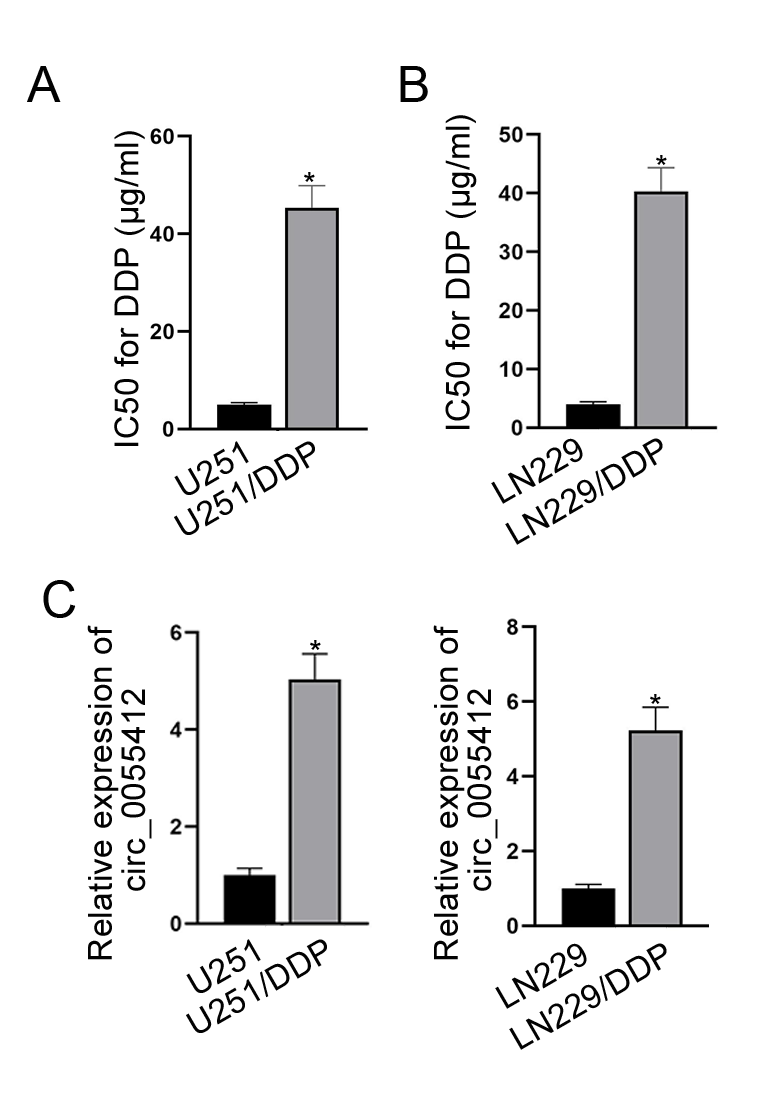

Supplement: Supplementary file 1 — Supplementary Material [file CNS-28-884-s001.zip › cns13820-sup-0001-FigS1.tif]

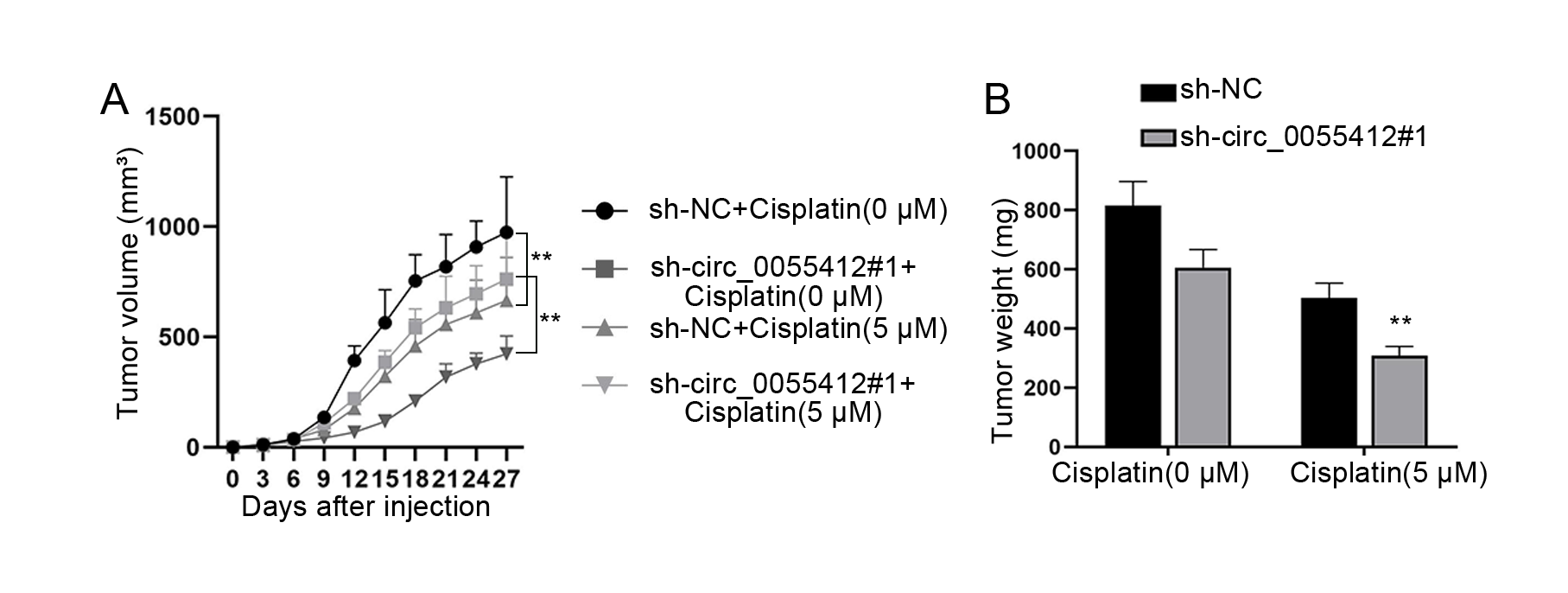

Supplement: Supplementary file 1 — Supplementary Material [file CNS-28-884-s001.zip › cns13820-sup-0003-FigS2.tif]

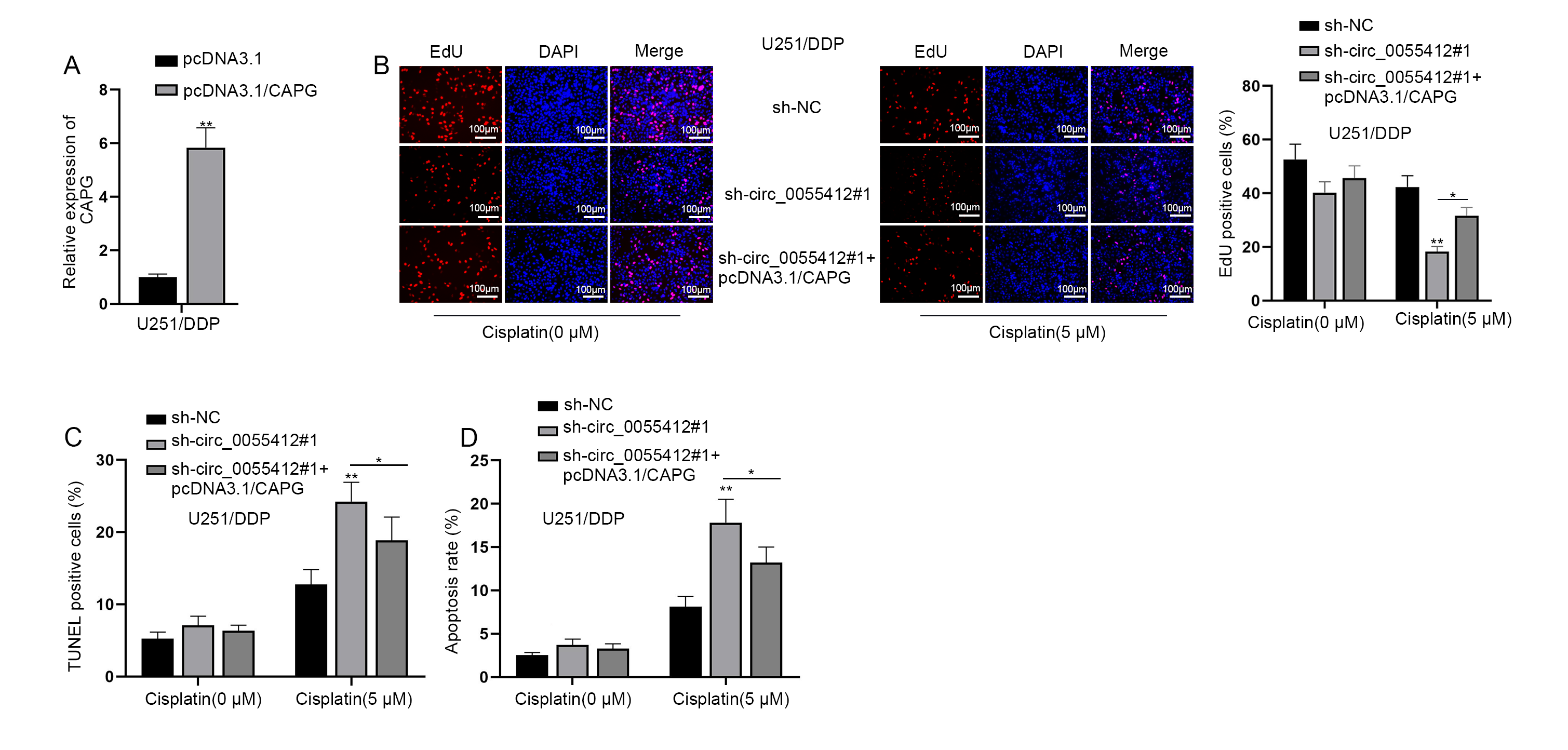

Supplement: Supplementary file 1 — Supplementary Material [file CNS-28-884-s001.zip › cns13820-sup-0004-FigS3.tif]

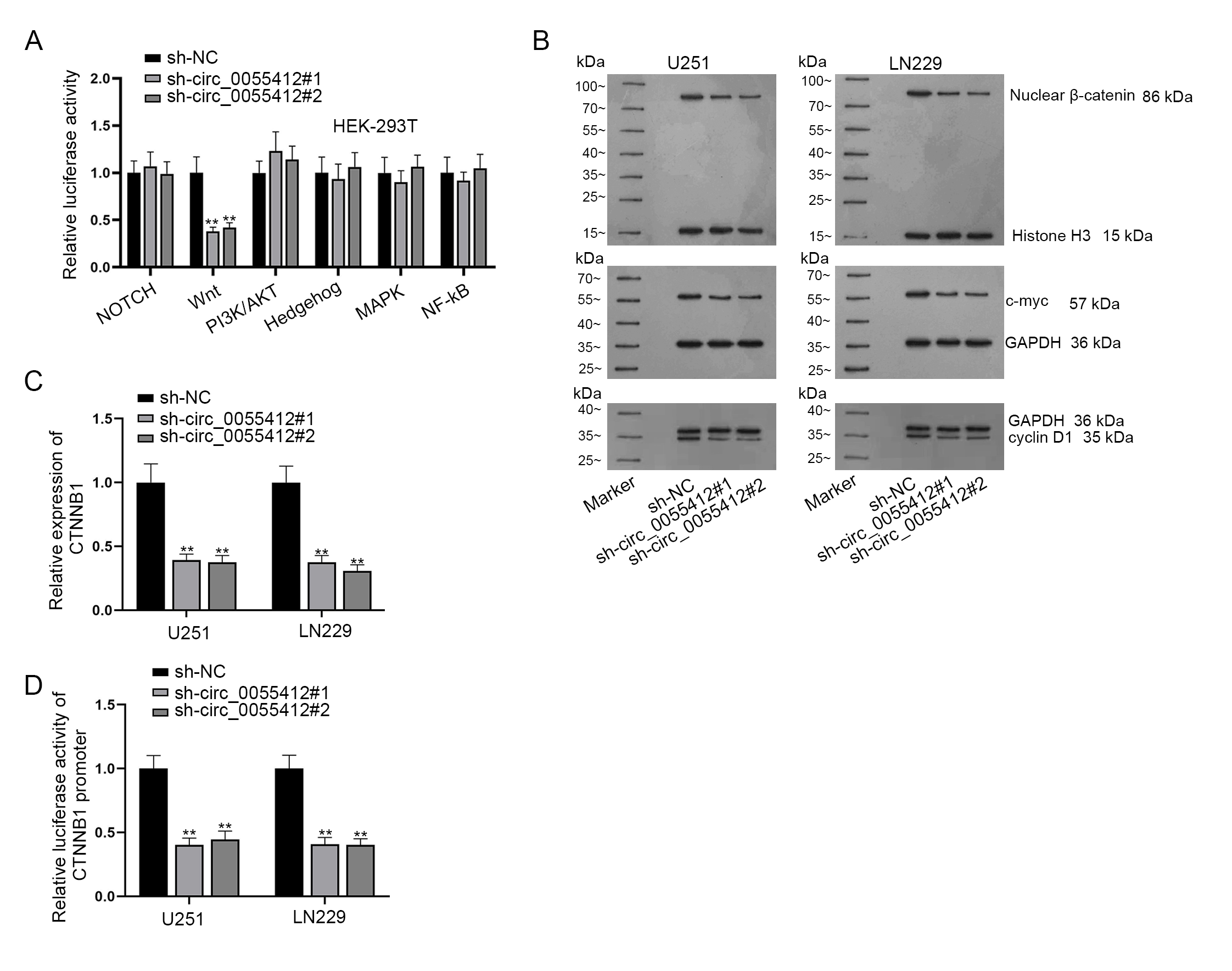

Supplement: Supplementary file 1 — Supplementary Material [file CNS-28-884-s001.zip › cns13820-sup-0005-FigS4.tif]

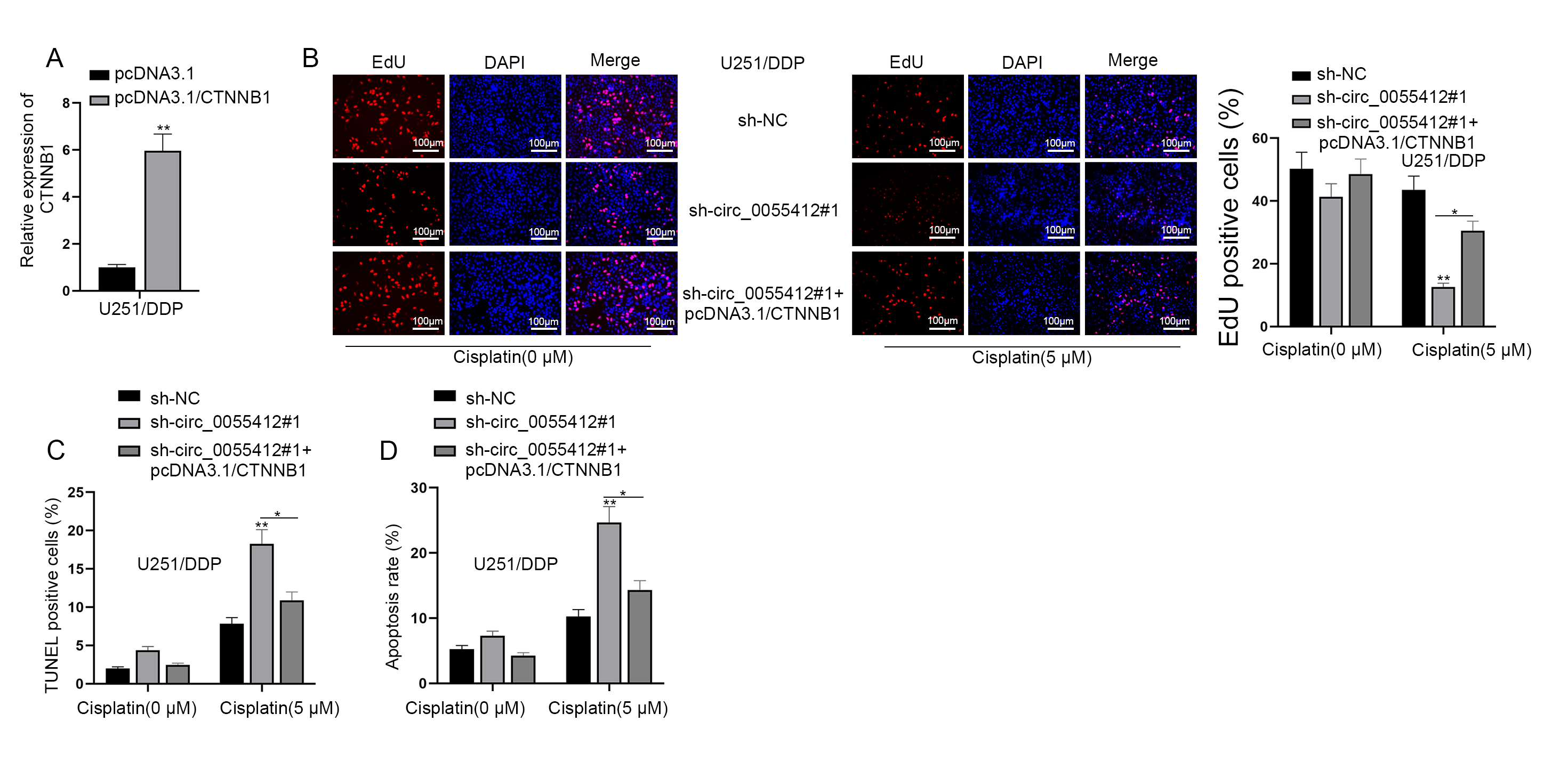

Supplement: Supplementary file 1 — Supplementary Material [file CNS-28-884-s001.zip › cns13820-sup-0006-FigS5.tif]
